# Relationship between the rate of change in lamina cribrosa depth and the rate of retinal nerve fiber layer thinning following glaucoma surgery

**DOI:** 10.1371/journal.pone.0206040

**Published:** 2018-11-06

**Authors:** Patrycja Krzyżanowska-Berkowska, Karolina Czajor, Iwona Helemejko, D. Robert Iskander

**Affiliations:** 1 Department of Ophthalmology, Wroclaw Medical University, Wroclaw, Poland; 2 Department of Biomedical Engineering, Faculty of Fundamental Problems of Technology, Wroclaw University of Science and Technology, Wroclaw, Poland; University of Houston, UNITED STATES

## Abstract

**Purpose:**

To assess whether lamina cribrosa depth (LCD) reduction and the rate of change in LCD over time (ΔLCD/Δt) is associated with retinal nerve fiber layer (RNFL) thickness and the rate of RNFL thinning over time (ΔRNFL/Δt) to test the hypothesis that, in a long term, RNFL thinning occurs irrespectively to the displacement of the lamina cribrosa following glaucoma surgery.

**Methods:**

Twenty-nine primary open-angle glaucoma patients underwent glaucoma surgery. Sixteen patients underwent trabeculectomy and 13 patients undertook non-penetrating deep sclerectomy. Images of optic nerve head using spectral-domain optical coherence tomography (SD-OCT) with enhanced depth imaging technology were obtained preoperatively, at one-, three-, six-month and follow-up postoperative visit from 12 to 29 months after surgery (1pv, 3pv, 6pv, and FUpv, respectively). Correspondingly, measurements of the circumpapillary RNFL thickness were acquired.

**Results:**

Intraocular pressure decreased from 24.0±8.9 to 10.9±3.9mmHg at 6pv (P<0.001) and to 12.7±4.4mmHg at FUpv (P<0.001). LCD was reduced from 465.3±136.4μm to 402.9±126.4μm at 1pv (P<0.001) and maintained similar position at 6pv (394.3±118.4μm; P = 0.170 with respect to 1pv). A significant decrease in the LCD was noted at FUpv (342.8±90.3μm, P<0.001) with respect to 6pv. RNFL thickness increased significantly to 64.9±19.8μm at 1pv (P = 0.005) and subsequently decreased to baseline level at 3pv. Further statistically significant decrease in RNFL thickness with respect to previous visit was found at 6pv and at FUpv (56.4±15.6μm and 55.0±14.0μm, P = 0.023 and P = 0.045, respectively). A thinner RNFL thickness at FUpv was not related to the LCD at FUpv (P = 0.129) but was correlated with ΔLCD/Δt at FUpv (P = 0.003). The ΔRNFL/Δt at FUpv was statistically significantly correlated with ΔLCD/Δt at FUpv (P<0.001).

**Conclusions:**

This is the first study that considers direct correlation between the rate of change in LCD with the rate of RNFL thinning over time. A thinner RNFL thickness following glaucoma surgery was associated with the rate of LCD reduction, not with position of the lamina cribrosa at the FUpv.

## Introduction

Glaucoma is a chronic, progressive optic neuropathy, in which the degeneration of retinal ganglion cells and corresponding visual field defect occur. Lamina cribrosa (LC) is believed to be the site of injury to retinal ganglion cell axons in glaucoma and recent advances in optical coherence tomography (OCT) have made it possible to visualize the posterior eye *in vivo*, including the LC [1,2]. One of the main concerns is the effect of IOP changes on the axons that pass through the laminar pores. Histologic studies demonstrated that compression and posterior deflection of the LC may cause damage to the axons and blockage of the axon flow [3].

Currently, the only known treatment for primary open angle glaucoma (POAG) is lowering of intraocular pressure (IOP) to slow down the progression of disease. Medical [4–6] or surgical [7–11] IOP reduction causes anterior displacement of the LC in the majority of POAG eyes. Taking into account the contribution of LC deformation in glaucomatous optic neuropathy, it may be hypothesized that release of pressure on the nerve fibers passing through the LC should also result in changes in RNFL thickness after IOP reduction. While there are numerous studies reporting the increased [12–14] or unchanged [15–17] RNFL thickness measured with OCT following glaucoma surgery, longitudinal studies that considered the relationship between RNFL thickness and LC position postoperatively are scanty [9,10]. We have recently shown that the magnitude of LC displacement was associated with significant, focal RNFL thinning at six months post-operatively [18]. This finding was in contrary to the assumption that sustained LCD reduction should be associated with a slow rate of disease progression after trabeculectomy [9]. If the RNFL thinning may occur despite the reduction of the LCD postoperatively, perhaps not only the change in the LC position is important after IOP lowering, but also the dynamics of this change over time.

The purpose of this study was to test the hypothesis that in a long term the RNFL thinning occurs irrespectively to the displacement of the LC, following glaucoma surgery. For that, the objective was to determine whether the LCD reduction and the rate of change in LCD over time (ΔLCD/Δ*t*) after surgery is associated with the retinal nerve fiber layer (RNFL) thickness and the rate of RNFL thinning over time (ΔRNFL/Δ*t*).

## Methods

### Study subjects

This is a prospective study that included 34 Caucasian subjects (17 male and 17 female) with POAG, who required glaucoma surgery because of a substantial increase in IOP, patients with glaucomatous progression confirmed with the visual field or optic disc examination despite maximally tolerated therapy or patients with advanced glaucoma [19]. Patients were scheduled for glaucoma surgery by two of the co-authors (PK-B and IH). Patients were enrolled from the Glaucoma Clinic at the Department of Ophthalmology, Wroclaw Medical University and were followed up from 12 to 29 months after surgery. The study was approved by the Bioethical Committee of the Wroclaw Medical University (No 345/2014) and adhered to the tenets of the Declaration of Helsinki. Informed written consent to participate was obtained from all subjects after the goals of the research and consequences of participation had been discussed.

All subjects underwent comprehensive ophthalmic examination and standard automated perimetry (Humphrey Field Analyzer II 750; 24–2 SITA-FAST; Carl Zeiss Meditec, Inc., Dublin, Ca). IOP measurements made by using Goldmann applanation tonometry were recorded at each visit. The inclusion criteria for patients consisted of the following: a diagnosis of POAG, a best corrected visual acuity of ≥ 20/40, spherical refraction of −3 to +3 diopters, and cylinder correction within ± 3.0 diopters. POAG was defined as the presence of glaucomatous optic nerve damage (i.e., concentric enlargement of the optic disc, presence of focal thinning, or notching) with associated visual field defects in the presence of an open angle. Subjects were excluded if they had a history of ocular surgery or laser treatment within 12 months before the onset of the study. Patients younger than 40 years old, those not being able to cooperate during the visual field tests, with intraocular disease (e.g., diabetic retinopathy, retinal vein occlusion) or neurological disorders affecting visual fields were also excluded from the study.

### Analysis of the retinal nerve fiber layer thickness

**Fig 1:**
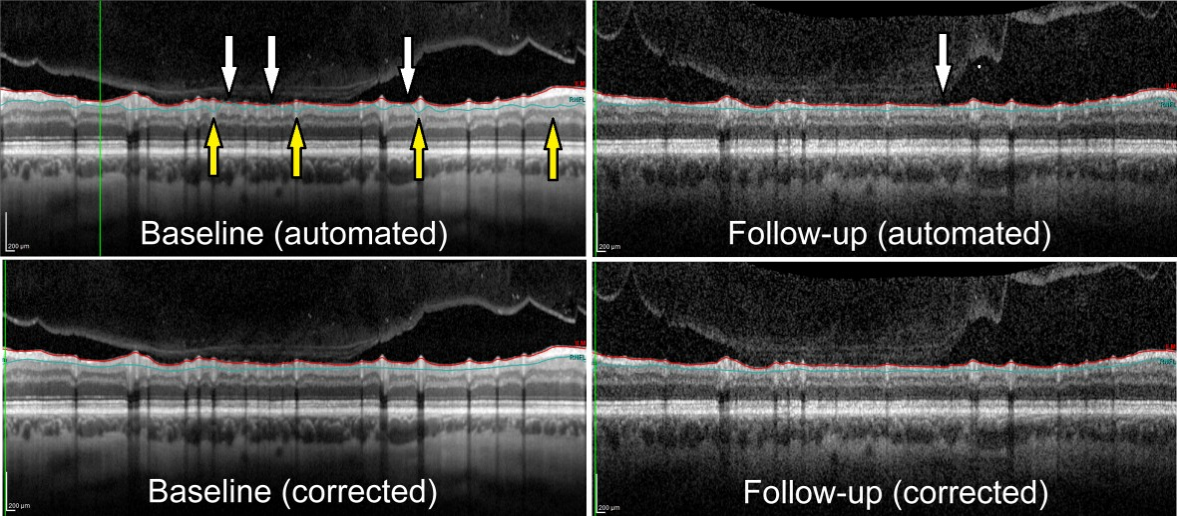
Examples of the extent of manual correction (refinement) of the automated segmentation necessary for adequate delineation of RNFL. White and yellow arrows denote the places where automated segmentation underestimates and overestimates the RNFL thickness, respectively.

The RNFL thickness was measured by SD-OCT (Spectralis, Heidelberg Engineering GmbH, Heidelberg, Germany) before surgery, at 1-month postoperative visit (1pv), 3-month postoperative visit (3pv) and 6-month and Follow-up postoperative visit (6pv and FUpv) using a circular scan protocol of the Spectralis OCT system. Follow-up scans were obtained using automated realignment procedure. The accuracy of the results was reviewed at each visit and segmentation errors were manually corrected [20]. Examples of the extent of such correction are shown in Fig 1. Only images that were considered appropriate by two independent observers (PK-B, KC) were included in the analysis. Impact of normal aging on the rate of RNFL thinning over time (ΔRNFL/Δ*t*) has been considered at each pv. The estimated average RNFL thinning related to the normal ageing process was assumed as -0.54 ± 0.23 μm/year [21]. Based on this definition a change in RNFL thickness of less than -0.54–1.96 × 0.23 μm/year was used to confirm thinning of the RNFL due to glaucoma.

### Image acquisition protocol

Serial horizontal B-scan images of the lamina cribrosa were obtained using Spectralis OCT system preoperatively, at 1pv, 3pv, 6pv and FUpv. The OCT device was set to image a 15 × 10 rectangle centered on the optic disc using the EDI technique. This rectangle was scanned with approximately 75 B-scan section images that were separated by 30 to 34 μm (the scan line distance was determined automatically by the instrument). Approximately 42 SD-OCT frames were averaged for each section. The SD-OCT images were acceptable for the study only when the quality score was higher than 18. For follow-up measurements set of B-scans were selected to correspond with preoperative images (follow-up mode).

### Analysis of the lamina cribrosa depth

All image processing procedures have been custom written in Matlab (MathWorks, Inc., Natick, MA, USA). Two points characterizing the Bruch’s membrane opening (BMO) and eight points describing the anterior LC were manually marked by an experienced operator (PK-B) for each OCT image using a specially designed graphic user interface. From that, the LCD was automatically calculated as a maximum perpendicular distance (corresponding to maximally depressed point) between the points of anterior LC surface and the line joining the two points of the BMO, referred further as the BMO line. The mean LCD was determined by averaging results from 12 to 16 individual central B-scans, where all considered points could be manually annotated without doubt. The number of scans, chosen equidistantly, depended on the size of the optic disc and was selected in a manner to cover up to three quarters of the optic disc.

**Fig 2:**
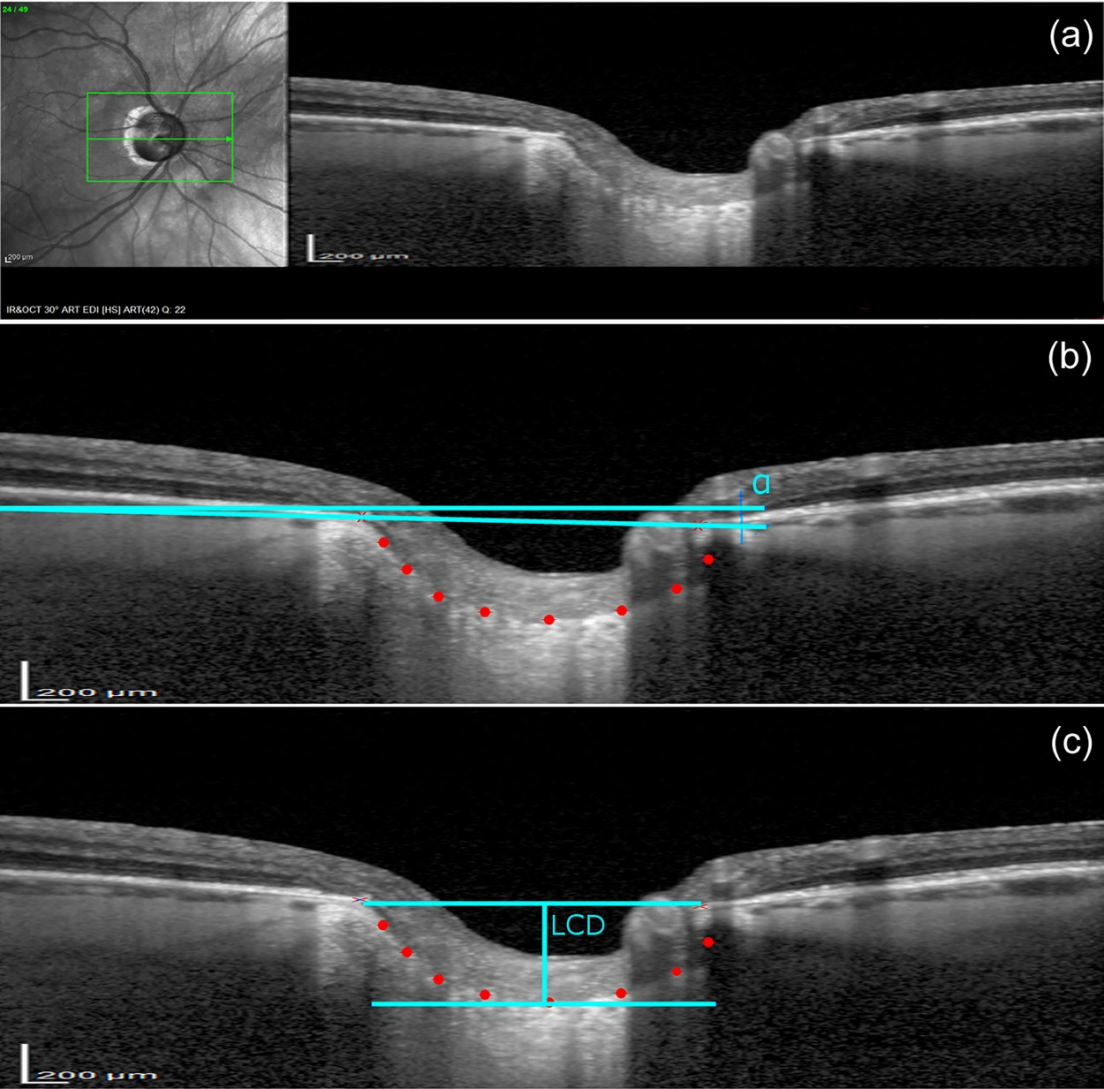
An example of infrared fundus photography and a B-scan image obtained at baseline and methods for determination of lamina cribrosa (LC). (a) An example of the acquired OCT image; (b) A reference line at an angle α to the horizontal line was set by connecting two points (red crosses) characterizing the Bruch’s membrane opening (BMO). Eight points describing the anterior LC surface (red dots) were manually placed using a specially designed graphic user interface; (c) An image rotated by α. The LC depth (LCD) was automatically calculated as the maximum perpendicular distance (corresponding to maximally depressed point) between the points of anterior LC surface and the BMO line.

Fig 2 shows an example of B-scan images with the manually selected points and automatically estimated LC depth.

### Numerical and statistical analysis

The rate of change in LCD over time as well as the rate of RNFL thinning over time were calculated, individually for each subject, using the ratios of the parameter differences between the postoperative visits and the baseline to the corresponding differences in time expressed in the units of years. Additionally, the rates of change of LCD and RNFL thinning were calculated over the period spanning between the 6pv and FUpv. Standard descriptive statistics were used along the Wilcoxon and Mann-Whitney tests, which were used to test for differences within and between the groups. Acknowledging that observations from the same subject are correlated, linearly mixed effect (LME) model was used to assess the LCD and RNFL changes over time. For example, the LCD the model is:

$${LCD}_{ij}=(\beta_{0}+b_{0i})+\beta_{1}T_{ij}{+\varepsilon }_{ij}$$

where subscript *i* denotes the subject, *j* indicates the time of measurement, while 0 and 1 indicate the intercept and the estimated slope, respectively. Constants *β* and *b* represent the fixed effects and random effect corresponding to each patient, respectively while *ε* denotes the random error. Similar LME models were considered for IOP and RNFL. This was followed by a step by step detailed analysis of changes occurring at each postoperative stage. Additionally, both univariate and multivariate linear regression was used to determine factors associated with the IOP reduction, change in the LCD and RNFL thickness. For multivariate analyses, a stepwise regression model was considered. Statistical analyses were performed using SPSS Statistics, version 22 (SPSS, Inc. Chicago, IL) and with Matlab. Unless otherwise stated, the value of *P* < 0.05 was considered significant.

## Results

The study included 34 POAG patients aged from 43 to 83 years (mean ± standard deviation (SD): 65.8 ± 10.8). Eighteen patients underwent trabeculectomy while 16 patients non-penetrating deep sclerectomy (NPDS). Our patients were all Caucasians and were matched for age between the groups and there were no statistically significant differences in visual field deterioration, central corneal thickness, OCT RNFL parameters and the number of glaucoma medications. The only statistically significantly different parameter was the baseline IOP (*P* = 0.006). The baseline data are presented in Table 1.

**Table 1:** Baseline data.

Baseline variables		Trabeculectomy	NPDS	*P* value*
Number of subjects (M/F)		16 (7/9)	13 (8/5)	-
Mean age (years ± SD) (range)		65.4 ± 10.1 (43–79)	66.3 ± 11.4 (53–83)	0.416
Mean CCT (μm ± SD) (range)		531 ± 30 (469–605)	528 ± 18 (486–547)	0.361
Mean AL (mm ± SD) (range)		23.47 ± 1.36 (21.15–25.96)	23.49 ± 0.75 (21.98–24.61)	0.547
Mean IOP (mmHg ± SD) (range)		27.4 ± 10.3 (16–56)	19.9 ± 4.0 (15–26)	**0.006**
Mean VF MD (dB ± SD) (range)		-15.32 ± 11.03 (-1.0 to -30.45)	-14.72 ± 10.76 (-2.98 to -28.80)	0.441
Mean VF PSD (dB ± SD) (range)		6.23 ± 3.58 (1.6–13.4)	6.45 ± 2.88 (2.6–13.4)	0.426
Average RNFL thickness (μm ± SD) (range)		55.75 ± 11.8 (37–81)	63.69 ± 19.2 (39–100)	0.159
Number of medications		3.4 ± 0.8 (2 to 4)	2.9 ± 0.7 (2 to 4)	0.110

NPDS = non-penetrating deep sclerectomy; M = male, F = female; SD = standard deviation; CCT = central corneal thickness; IOP = intraocular pressure; VF MD = visual field mean deviation; VF PSD = visual field pattern standard deviation; RNFL = retinal nerve fiber layer; values with statistical significance are shown in bold

* Mann-Whitney rank sum test

Four patients were lost to follow-up shortly after surgery and one patient was lost after six months. The remaining 29 patients were followed up at one, three, six months and from 12 to 29 months (mean±SD: 19.7 ± 4.0) after surgery. It is worth noting that among all patients included in the study only 3 subjects had follow-up period of 12 months. Most of the patients had an observation period of 20 months or more. The raw data are collated in S1 Table.

**Fig 3:**
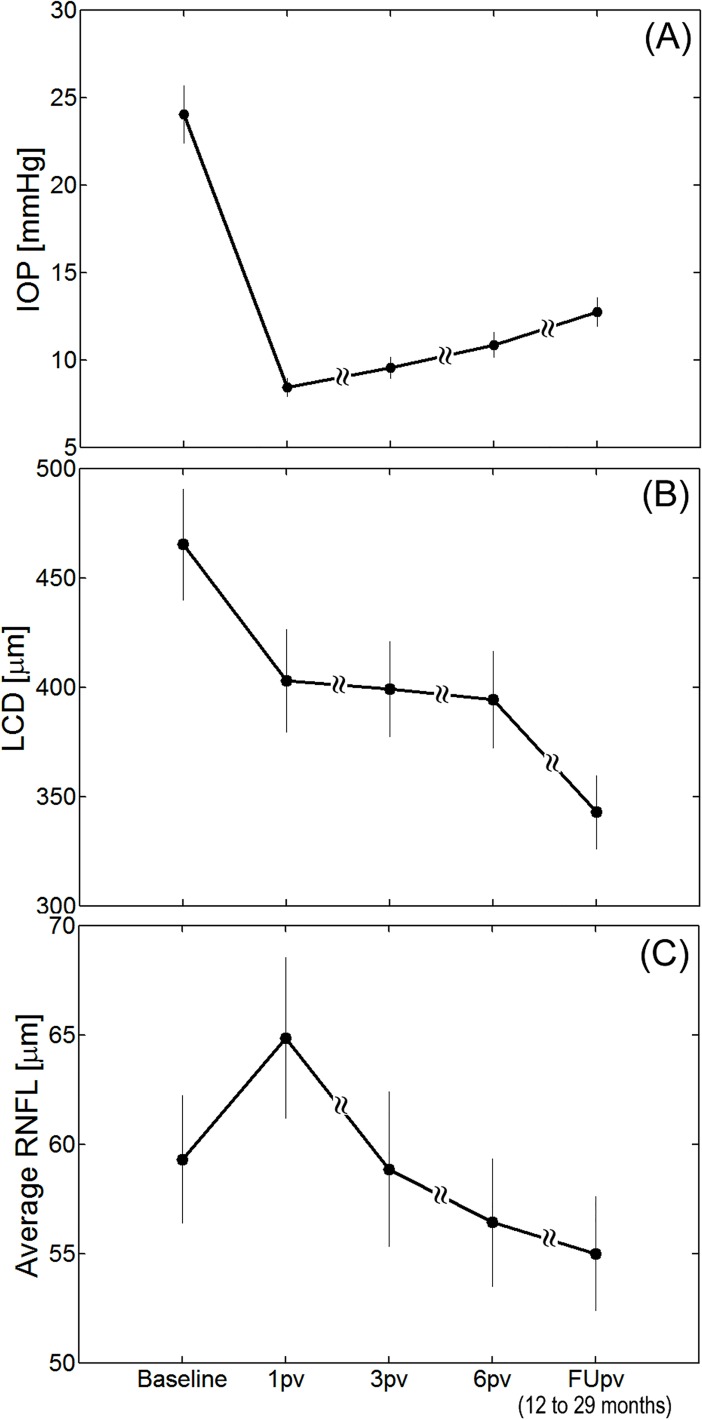
The time course of the intraocular pressure (IOP) (A), lamina cribrosa depth (LCD) (B) and average RNFL thickness (C) at each control visit with respect to the baseline values. The error bars denote one standard error. Note that the points on the abscissa are not equidistant.

The IOP decreased significantly from 24.0 ± 8.9 preoperatively to 8.41 ± 2.8 mmHg at 1pv (Wilcoxon test, *P* < 0.001), 9.6 ± 3.3 mmHg at 3pv (*P* < 0.001), 10.9 ± 3.9 mmHg at 6pv (*P* < 0.001) and 12.7 ± 4.4 mmHg at FUpv (*P* < 0.001). See Fig 3A.

The results of the LME model for the IOP change over time revealed an intercept *β*_0_ = 13.93 (CI: 12.35; 15.51) mmHg and a nonsignificant slope *β*_1_ = −1.63 (CI: -3.63; 0.37) mmHg/year (*P* = 0.111), indicating that a linear model is not necessarily well representing the nature of IOP change over time (see Fig 3A). For LCD change over time, the LME model showed statistical significant intercept *β*_0_ = 427.6 (CI: 383.2; 471.9) μm and a slope *β*_1_ = −53.2 (CI: -69.6; -36.7) μm /year (both *P* < 0.001). Similarly, for RNFL change over time, the LME model showed statistical significant intercept *β*_0_ = 60.8 (CI: 54.5; 67.0) μm and a slope *β*_1_ = −3.8 (CI: -5.7; -1.8) μm /year (both *P* < 0.001).

Considering detailed analysis, the average rate of IOP reduction was first at -187.4 mmHg/year at 1pv (i.e., a reduction of 187.4/12 = 15.61 mmHg in the first month postoperatively) and then decreased significantly to -57.9, -26.3, and -7.9 mmHg/year (all *P* < 0.001 at 3pv, 6pv and FUpv, respectively). Interestingly, there were statistically significant differences between both IOP levels as well as IOP rates between 6pv and FUpv (*P* = 0.008 and *P* < 0.001, respectively).

The lamina cribrosa depth (LCD) decreased significantly from 465.3 ± 136.4 μm preoperatively to 402.9 ± 126.4 μm at 1pv (*P* < 0.001), and maintained a similar, anterior position at 3pv (399.2 ± 116.9 μm; *P* = 0.421) and at 6pv (394.3 ± 118.4 μm; *P* = 0.170) with respect to the value at the first month after surgery. At the FUpv, we found further statistically significant reduction of LCD to 342.8 ± 90.3 μm (*P* < 0.001) with respect to the value at 6pv. See Fig 3B. The average rate of LCD reduction was first at -748.6 μm/year at 1pv (i.e., a reduction of 748.6 /12 = 62.38 μm in the first month postoperatively), and then decreased significantly to -264.5, -141.9, and -75.3 μm/year (all *P* < 0.001 at 3pv, 6pv and FUpv, respectively). There was a statistically significant difference between the rates of LCD reduction at 6pv and FUpv (*P* = 0.007). The Mann-Whitney test showed that there were no statistically significant differences in LCD rates between the procedures at any postoperative visit (*P* = 0.809, *P* = 0.913, *P* = 0.983, *P* = 0.142, at 1pv, 3pv, 6pv and FUpv, respectively). Concerning the LCD reduction in detail, five eyes exhibited statistically insignificant increase of the LCD at 1pv (Wilcoxon test, *P* = 0.063), ranging from 4 μm to 57 μm and then decrease at 3pv (*P* = 0.125).

The average RNFL thickness was 59.3 ±15.8 μm before surgery and increased significantly to 64.9 ±19.8 μm at 1pv (*P* = 0.005) and subsequently decreased to 58.9 ±19.0 μm at 3pv (*P* = 0.205) with respect to the value at the baseline. At the 6pv and FUpv, we found further statistically significant decrease of the average RNFL thickness to 56.4 ± 15.6 μm (*P* = 0.023) with respect to the value at 3pv and to 55.0 ± 14.0 μm (*P* = 0.045) with respect to the value at 6pv, respectively. See Fig 3C.

The ΔRNFL/Δ*t* results are shown in Table 2 where the effect of aging process on the statistics (last column) is taken into account. It was found that between 6pv and the FUpv there was a statistically significant difference in ΔRNFL/Δ*t* (*P* = 0.033). Taking into account the effect of natural aging at FUpv eight subjects had no change or had improvement in the average RNFL thickness where the rate of change was 2.1 ± 1.9 μm/year (range -0.6 to 5.1). Subsequently, in this part of data analysis patients were divided into 2 groups: 1) progressive group of 21 subjects who showed statistically significant RNFL thinning exceeding thinning RNFL rates that could be age related (see the last column of Table 2 where the results of the Wilcoxon test for that group is provided between 6pv and FUpv) and 2) non-progressive group of 8 subjects who showed no change or had improvement of RNFL thickness. At the baseline, there was no statistically significant difference in age between these two groups (*P* = 0.235), but there was a statistically significant difference in LCD (544 ± 148 μm for non-progressive group and 435 ± 122 μm for progressive group, *P* = 0.045) and RNFL (50 ± 6 μm for non-progressive group and 63 ± 17 μm for progressive group, *P* = 0.002). In the non-progressive group, 6 eyes underwent trabeculectomy and 2 eyes NPDS, while in the progressive group, 10 eyes underwent trabeculectomy and 11 eyes NPDS.

**Table 2:** The rate (speed) of retinal nerve fiber layer thinning over time. The units are μm per year. Positive values correspond to thickening while negative values correspond to thinning.

RNFL location	Over 1 month	Over 3 months	Over 6 months	Over all period until Follow-up	FU vs. 6M *(P*)*	FU vs. 6M (*P*)**
Average	66.6 ± 113.0 (-84.0, 396.0)	-1.8 ± 35.6 (-44.0, 108.0)	-5.8 ± 13.1 (-30.0, 34.0)	-2.8 ± 4.0 (-12.6, 5.1)	**0.033**	**<0.001**
Temporal Superior	31.4 ± 120.4 (-192.0, 432.0)	-6.9 ± 30.7 (-76.0, 44.0)	-4.5 ± 14.4 (-32.0, 32.0)	-4.1 ± 5.7 (-16.0, 4.4)	0.642	0.099
Temporal	45.9 ± 102.5 (-120.0, 372.0)	-3.9 ± 28.4 (-60.0, 68.0)	-3.2 ± 12.5 (-28.0, 28.0)	-1.4 ± 3.9 (-7.4, 8.6)	0.182	0.070
Temporal Inferior	-5.4 ± 115.3 (-216.0, 300.0)	-17.0 ± 51.7 (-156.0, 144.0)	-11.6 ± 20.9 (-74.0, 24.0)	-4.6 ± 7.7 (-33.1, 6.9)	**0.031**	**0.004**
Nasal Superior	74.5 ± 141.7 (-108.0, 492.0)	7.3 ± 43.9 (-108.0, 136.0)	-5.0 ± 25.2 (-106.0, 38.0)	-3.2 ± 7.3 (-29.3, 9.0)	0.750	0.100
Nasal	40.6 ± 107.3 (-132.0, 300.0)	-3.2 ± 27.0 (-40.0, 72.0)	-4.5 ± 15.25 (-32.0, 40.0)	-2.1 ± 4.2 (-8.3, 7.6)	0.142	**0.002**
Nasal Inferior	67.4 ± 204.9 (-192.0, 948.0)	-0.8 ± 48.3 (-64.0, 120.0)	-9.0 ± 17.8 (-40.0, 32.0)	-3.7 ± 6.5 (-21.1, 10.7)	**0.050**	**0.002**

Data are presented as mean ± standard deviation and the range in brackets. FU–follow-up. 6M – 6 months.

Values with statistical significance are shown in bold

* Wilcoxon sign rank test for the whole group (29 subjects)

** Wilcoxon sign rank test for the group of 21 progressive subjects exceeding thinning RNFL rates that could be age related [21]

Also, the following sectors of RNFL thickness were analyzed: temporal superior (TS), temporal (T), temporal inferior (TI), nasal superior (NS), nasal (N) and nasal inferior (NI). Table 2 shows that in TI and NI sectors there was thinning exceeding the natural aging process at FUpv and this thinning increased statistically significantly in relation to 6pv (*P* = 0.031 and *P* = 0.050, for TI and NI respectively). Analysis of the progressive group with confirmed RNFL thinning showed lower levels of *P* values in those sectors (*P* = 0.004 for TI and *P* = 0.002 for NI) and extended the results to sector N (*P* = 0.002).

Univariate correlation analysis was performed between all IOP, LCD and RNFL measurements, their rates and age.

Subsequently, for further multivariate analysis only those parameters that resulted in statistically significant correlation (*P* < 0.10) as well as not being functionally dependent were taken into account (see Table 3, where only those correlation results that were statistically significant are listed).

**Table 3:** Results of univariate and multivariate linear regression analyses to determine factors associated with the IOP reduction, change in the LCD and RNFL thickness. Listed are only those variables that achieved univariate correlation at the significance level of ≤ 0.1.

Correlates		Univariate analysis				Multivariate analysis
Variable 1	Variable 2	β	95% Confidence Interval	*R*	*P*	*P*
RNFL base	LCD base	–4.22	–7.20 to –1.24	–0.488	**0.007**	‘In’, **0.007**
	LCD rate 1	0.0085	0.0012 to 0.016	0.416	**0.025**	‘Out’, 0.145
RNFL 1pv	LCD base	–0.054	–0.107 to –0.0004	–0.370	**0.048**	‘Out’, 0.214
	LCD rate 1pv	0.011	0.0021 to 0.0202	0.438	**0.017**	“In’, **0.018**
RNFL 3pv	LCD base	–0.056	–0.106 to –0.0051	–0.399	**0.032**	‘In’, **0.032**
	LCD rate 3pv	0.017	–0.0029 to 0.037	0.320	0.091	‘Out’, 0.477
RNFL 6pv	LCD base	–0.058	–0.097 to –0.018	–0.503	**0.005**	‘Out’, 0.179
	LCD rate 6pv	0.054	0.028 to 0.080	0.635	**<0.001**	‘In’, **<0.001**
RNFL FUpv	LCD base	–0.048	–0.084 to –0.013	–0.472	**0.010**	‘Out’, 0.291
	LCD rate 1pv	0.0098	0.0038 to 0.016	0.544	**0.002**	‘Out’, 0.390
	LCD rate 3pv	0.017	0.026 to 0.031	0.424	**0.022**	‘Out’, 0.208
	LCD rate 6pv	0.049	0.026 to 0.072	0.647	**<0.001**	‘In’, **<0.001**
	LCD rate FUpv	0.134	0.049 to 0.220	0.527	**0.003**	‘Out’, 0.351
RNFL rate FUpv–6pv	LCD base	0.011	0.0007 to 0.021	0.388	**0.037**	‘Out’, 0.946
	LCD rate FUpv	–0.043	–0.065 to –0.021	–0.613	**<0.001**	‘In’, **<0.001**
IOP base	age	–0.448	–0.721 to –0.174	–0.543	**0.002**	‘In’, **0.013**
	LCD rate 1pv	–0.004	–0.0084 to –0.000	–0.369	**0.049**	‘Out’, 0.777
	LCD rate 3pv	–0.009	–0.018 to 0.0001	–0.364	**0.052**	‘Out’, 0.743
	LCD rate 6pv	–0.017	–0.034 to 0.0012	–0.346	0.066	‘Out’, 0.889
	LCD rate FUpv	–0.088	–0.141 to –0.034	–0.542	**0.002**	‘In’, **0.013**

RNFL base = RNFL at the baseline; LCD base = lamina cribrosa depth at the baseline; 1pv, 3pv, 6pv–one, three, and six months post-operatively, respectively; FUpv–follow-up postoperative visit; FU–6pv = Follow-up– 6 months postoperative visit; β –slope; R–correlation coefficient; ‘In’ and ‘Out’ denotes the status of the variable and whether it is or it is not, included in the model.

Values with statistical significance are shown in bold

Univariate analysis revealed that a thinner RNFL thickness at FUpv was not related to the LCD at FUpv (*P* = 0.129), but was positively correlated with the LCD change rate at FUpv (*P* = 0.003). In multivariate analysis a thinner RNFL thickness at the 6pv and FUpv was positively correlated with the faster rate of LCD change at 6pv (*P* < 0.001 for both RNFL at 6pv and FUpv). During the observation period between 6 months and follow-up visit (FU– 6m) the faster rate of RNFL thinning at the final FUpv was statistically significantly negatively correlated with a faster change of LCD over time (ΔLCD/Δ*t*) at FUpv (*P* < 0.001).

**Fig 4:**
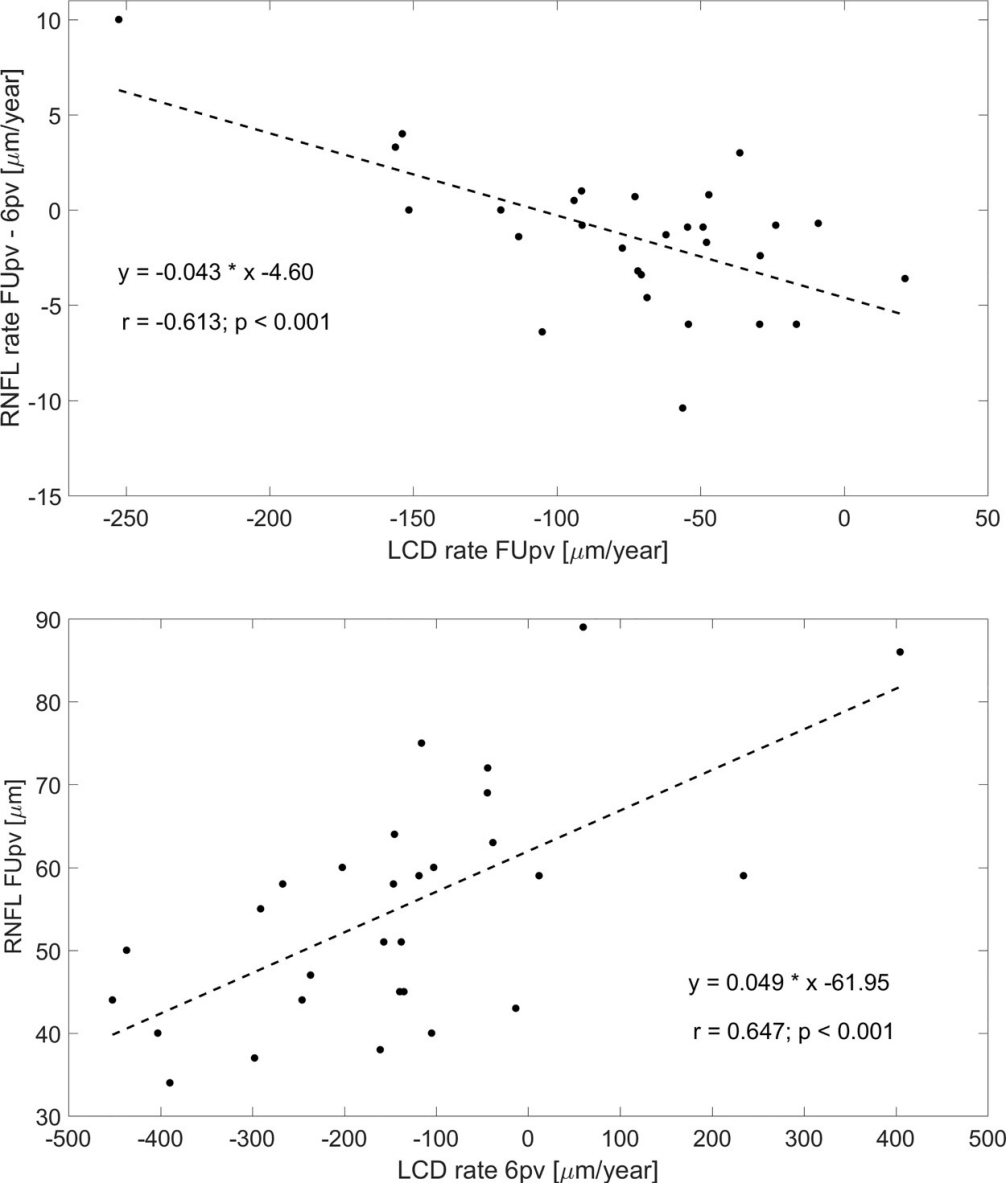
Correlations between RNFL rate at FUpv–6pv and LCD rate at FUpv (top plot) and between RNFL FUpv and LCD rate at 6pv (bottom plot).

Considering the LCD, univariate analysis revealed that a deeper LCD at the baseline was statistically significantly negatively correlated to the faster thinning of the RNFL thickness at all stages of the study, preoperatively (*P* = 0.007), at 1pv (*P* = 0.048), 3pv (*P* = 0.032), 6pv (*P* = 0.005) and FUpv (*P* = 0.010). The correlation between the average RNFL thickness and LCD at each postoperative visit was lost, at 1pv (*P* = 0.359), 3pv (*P* = 0.244), 6pv (*P* = 0.660) and FUpv (*P* = 0.129), but statistically significant positive correlation between the faster thinning of the RNFL thickness and the rate of change in LCD was found at each visit (Table 3). Multivariate analysis showed that a deeper LCD preoperatively was negatively correlated with a thinner RNFL thickness at the baseline (*P* = 0.007). Additionally, in Fig 4, correlation between RNFL rate at FUpv–6pv and LCD rate at FUpv as well as the correlation between RNFL FUpv and LCD rate at 6pv are shown in a graphical form.

The higher IOP preoperatively was negatively correlated with the faster rate of change in LCD at all stages of the study, at 1pv (*P* = 0.049), 3pv (*P* = 0.052), 6pv (*P* = 0.066) and FUpv (*P* = 0.002) in univariate analysis. In multivariate analysis only positive correlation between IOP at the baseline and the ΔLCD/Δ*t* at FUpv was statistically significant (*P* = 0.013). The significant correlation between the higher rate of IOP change and the faster rate of LCD change was found at 1pv (*P* = 0.032), 3pv (*P* = 0.026), 6pv (*P* = 0.062) and FUpv (*P* = 0.001)

There was also a statistically significant negative correlation between age and higher IOP preoperatively in univariate (*P* = 0.002) and multivariate model (*P* = 0.013).

### Representative cases

**Fig 5:**
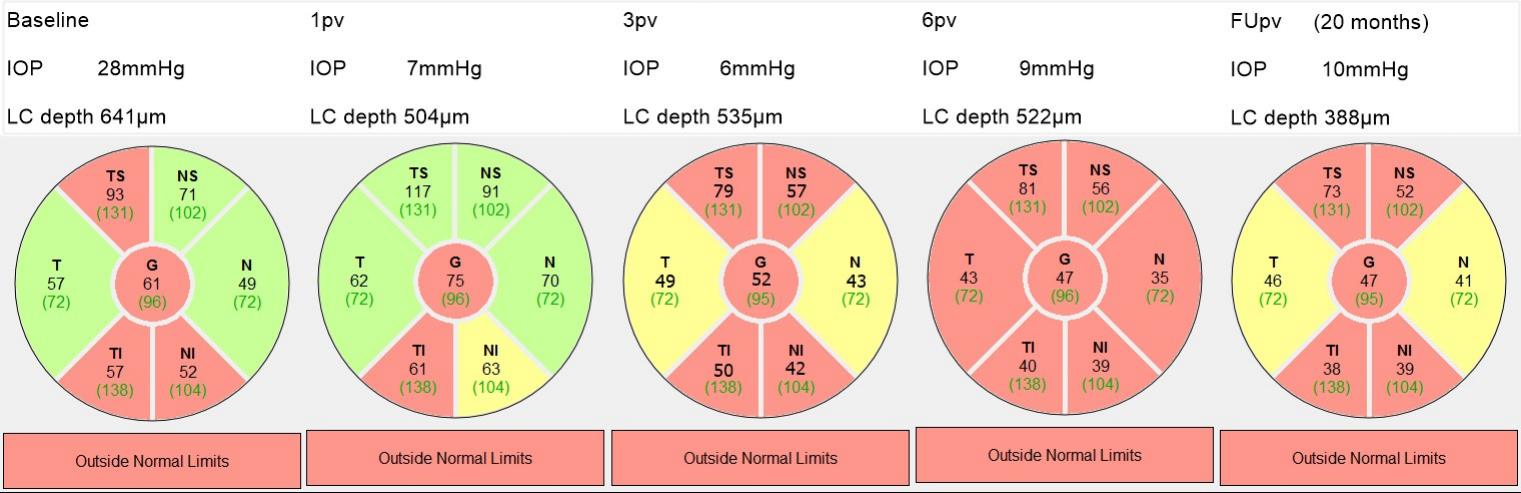
The time course of RNFL thinning with the corresponding IOP and LCD measurements for the right eye of a 62-year-old male patient. FUpv at 20 months. G = global, TS = temporal superior, T = temporal, TI = temporal inferior, NS = nasal superior, N = nasal, NI = nasal inferior.

**Fig 6:**
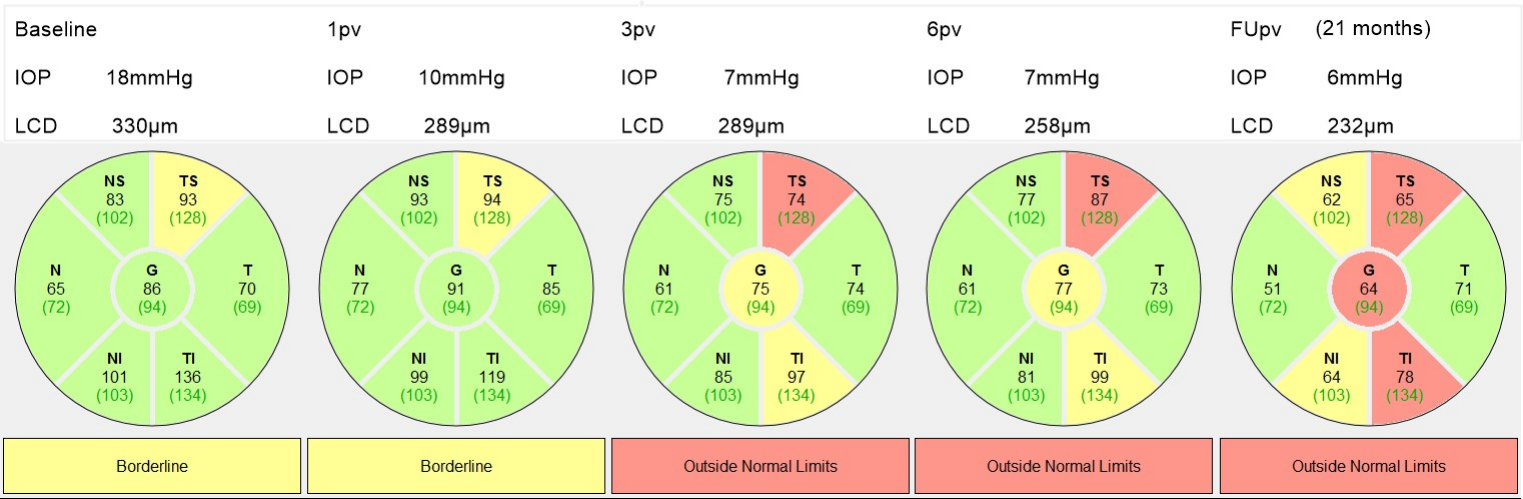
The time course of RNFL thinning with the corresponding IOP and LCD measurements for the left eye of an 81-year-old female patient. FUpv at 21 months. G = global, TS = temporal superior, T = temporal, TI = temporal inferior, NS = nasal superior, N = nasal, NI = nasal inferior.

Figs 5 and 6 show the results of RNFL thinning of two patients with different LCD reductions at the postoperative visits, different RNFL thickness at the baseline and after two different surgeries. Fig 5 shows a patient who underwent trabeculectomy with a substantial LCD reduction at 6pv (from 641 μm at the baseline to 522μm at 6 pv) and at FUpv (from 522 μm at 6pv to 388 μm at FUpv). Correspondingly, the average RNFL thickness decreased from 61 μm at the baseline to 47 μm at 6pv and remained at the same level at FUpv. The rate of RNFL thinning was -8.4 μm/year at FUpv, however, the bulk of the change in RNFL thickness occurred during the first six months. The observation period was 20 months.

Fig 6 shows a patient who underwent NPDS with smaller LCD reduction than that exhibited in the previous patient from 330 μm at the baseline to 258 μm at 6pv and a further change to 232 μm at FUpv. Correspondingly, the average RNFL thickness decreased from 86 μm at the baseline to 77 μm at 6pv and to 64 μm at FUpv. The rate of RNFL thinning was -12.6 μm/year at FUpv. The observation period was 21 months.

## Discussion

This is a longitudinal study that demonstrated the relationship between the LC position and RNFL thickness following glaucoma surgery. Although numerous studies evaluated the RNFL thickness after glaucoma surgery [12–17], reports on the rate of RNFL thickness loss and its relationship to the LC are scanty [9,10]. It has been reported that the LC could move anteriorly after glaucoma surgery [7–10]. This movement is potentially related to a relief of compressed axons as compared to the preoperative stage.

In the present study, apart from the substantial reduction of the LC within six months postoperatively, a significant decrease in the LCD was also noted at FUpv with respect to 6pv. This indicates continuing change of LC position in a view of stable IOP, being in agreement with recently reported data [10].

Our data showed swelling of the retinal nerve fibers one month after surgery and their return to the initial state after three months. This period was also associated with a substantial displacement of the LC anteriorly (Fig 3). Interestingly, although on average, the LC moved anteriorly over the whole course of observation, continuous thinning of the RNFL thickness was observed. These results were compared to those obtained by Lee and Kim [9] who presented the rate of RNFL thinning statistically significantly correlated with the LCD change between 6pv and FUpv. They found, however, that a larger increase in LCD between 6pv and FUpv was associated with a faster rate of RNFL thinning. By contrast, eyes for which the LCD reduction sustained until FUpv had a slower rate of progression. In the present study, we have not observed statistically significant posterior movements of LC in the period between 6pv and FUpv, but further decrease in LCD, accompanied by progressive RNFL thinning at that time. Continuing movement of the LC anteriorly may be due to possible postoperative changes in the mechanical properties of the posterior eye and the preservation of reduced IOP with respect to the pre-operative stage.

Our analysis of RNFL thinning also included the influence of normal aging in order to provide age-independent interpretation of RNFL progression [21]. Thus, progression was considered to exist when a significant negative trend for RNFL thickness measurements was present and exceed the values that could be age related. Based on this, we found progressive group of 21 patients with the baseline RNFL thickness values greater than those of the non-progressive group of eight subjects. Our results are consistent with other studies that reported glaucomatous patients with a thicker RNFL at baseline to have faster rate of progressive RNFL thinning [6, 22].

There is a recent study conducted in patients with acute primary angle-closure (APAC) that reported significant LCD reduction and the RNFL loss during a 12-month follow-up [23]. The authors speculate that significant displacement of the LC could be responsible for the axonal damage while insignificant LCD reduction after IOP lowering should be associated with the lack of RNFL loss. However, results of the present study do not fully support this supposition. As shown in Figs 5 and 6, the progressive RNFL loss at follow-up terms of 20 and 21 months, respectively, occurs irrespectively whether large or small LCD reduction is introduced. Our analysis revealed that LC at baseline is placed more anteriorly in the 21 progressive subjects than in the case of the eight non-progressive subjects. Hence, if the position of LC and the extent of its displacement following the surgery do not affect RNFL thinning, it could be speculated that it is important to determine the speed of LC displacement after the procedure.

An important finding in this study is that the faster rate of RNFL thinning at the final FUpv was strongly correlated with the faster rate of LCD reduction at FUpv. To the best of our knowledge this is the first study that considers direct correlation between the rate of change in LCD over time and the rate of RNFL thinning following glaucoma surgery.

It is generally known that the structure of lamina cribrosa is remodeled in glaucoma eyes, becomes deeper and wider under the influence of various factors including IOP [24, 25]. The aging process also alters the ocular mechanical response to IOP due to the stiffness of the sclera, redistribution of the collagen and reduction of pores for axons passing [26]. Based on the results of our research, we can hypothesize that a rapid change in IOP after surgery combined with the sudden change of LC position and the surrounding tissues could be detrimental to axons, which pass through the distorted LC pores.

Worth noting is that not only the change in the LC position is important after IOP lowering, but also the dynamics of this change over time. Our study revealed that eyes with the greater RNFL thickness loss at the final FUpv demonstrates faster rate of the LC movement in the first 6 months after surgery. Interestingly, a thinner RNFL thickness at FUpv was not related to the further LCD reduction at FUpv, but was correlated with the LCD change rate over time at FUpv. Also, it is important to note that although LCD rates were associated with baseline IOP and subsequently RNFL thinning rates were to be associated with LCD rates, this cannot be said for IOP rates versus RNFL thinning. We should emphasize that no statistically significant correlations were found between the IOP reduction and the RNFL thickness loss or the speed of the RNFL thinning. Perhaps, studies with less invasive surgical IOP reduction techniques could provide more insight into this mechanism.

Our study included patients who underwent two different procedures. Originally we compared these two groups and our results revealed that regardless of the performed procedure, statistically significant anterior displacement of LC was found at 6pv [18]. Note that in this study, similar LCD was observed at 6pv (389.4 ± 121.2 μm for trabeculectomy and 400.3 ± 109.8 μm for NPDS) and FUpv (324.6 ± 73.7 μm for trabeculectomy and 365.2 ± 99.8 μm for NPDS), with no statistically significant differences between the groups (*P* = 0.405 and *P* = 0.126 for trabeculectomy and NPDS, respectively). This observation is in opposition to Barrancos et al. [27], who suggest that in patients after NPDS early cupping reversal is mainly due to a postoperative increase in the prelaminar tissue.

This study has some limitations. First, the LC depth was measured from the BMO level, which is influenced by the choroidal thickness. It is well known that the change of the choroidal thickness after surgery would affect the LCD and may cause its overestimation postoperatively [28]. However, in our study the LCD was still decreased after surgery despite the possibility of overestimation, which in fact reinforces the results of the study. Second, the mean follow-up period was not uniform in all patients but the time of the FUpv was taken into account in calculating the rates of the considered parameters. Third, the mechanical properties of the ONH and PPS were not considered, however those parameters are not currently measurable.

In conclusion, this study provides evidence for further glaucoma progression in patients with POAG despite substantial IOP reduction and LCD decrease in a mid-term observation period. To the best of our knowledge, this is the first study that considers direct correlation between the rate of change in LCD with the rate of RNFL thinning following surgical IOP reduction. The rate of RNFL thinning over time was found to be associated with the rate of LCD reduction, but not with the position of the LC in a course of study.

## Supporting information

S1 Table The original raw data for the experiment as supplementary excel file. (XLSX)
